# Correlations between inflammatory cytokines, muscle damage markers and acute postoperative pain following primary total knee arthroplasty

**DOI:** 10.1186/s12891-017-1597-y

**Published:** 2017-06-17

**Authors:** Hai-bo Si, Ti-min Yang, Yi Zeng, Zong-ke Zhou, Fu-xing Pei, Yan-rong Lu, Jing-qiu Cheng, Bin Shen

**Affiliations:** 1 Department of Orthopedic Surgery, West China Hospital, Sichuan University, 37th Guoxue Road, Chengdu, Sichuan 610041 China; 2 Key Laboratory of Transplant Engineering and Immunology, West China Hospital, Sichuan University, No.1 Keyuan 4th Road, Chengdu, Sichuan 610041 China

**Keywords:** Total knee arthroplasty, Acute postoperative pain, Inflammatory cytokines, Muscle damage markers, Body mass index

## Abstract

**Background:**

Despite the success of total knee arthroplasty (TKA) in reducing knee pain and improving functional disability, the management of acute postoperative pain is still unsatisfactory. This study was aimed to quantitatively analyze the possible correlations between inflammatory cytokines, muscle damage markers and acute postoperative pain following primary TKA.

**Methods:**

Patients scheduled for unilateral primary TKA were consecutively included, the serial changes of the numerical rating scale (NRS) at rest (NRSR) and at walking (NRSW), serum inflammatory cytokines and muscle damage markers were assessed before surgery (T0) and at postoperative day 1, 2, 3 and 5 (T1-T4, respectively); while pain disability questionnaire (PDQ) and synovial fluid inflammatory cytokines were evaluated at T0. The correlations between inflammatory cytokines, muscle damage markers and pain scores were examined, and Bonferroni correction was applied for multiple comparisons.

**Results:**

Ninety six patients were included for serum markers and pain evaluations at T0-T4, while 54 (56.25%) for synovial fluid cytokines at T0. The NRSR at T1 and T2 were positively correlated with preoperative NRSW, while the NRSW at T1 to T4 were positively correlated with preoperative NRSR, NRSW and PDQ (all *p* < 0.05). The NRSR was positively correlated with serum PGE2, IL-6, and CK at T1; the NRSW was positively correlated with serum CRP at T1, with PGE2 and IL-6 at T1 to T3, with CK at T2 and T4, and with Mb and LDH at T1 to T4 (all *p* < 0.003). Meanwhile, positive correlations were observed between preoperative NRSW and synovial fluid PGE2, IL-6, IL-8, or TNF-α, as well as between PDQ and PGE2 (all *p* < 0.003), but no associations between postoperative pain scores and preoperative synovial fluid cytokines was found (all *p* ≥ 0.003). Additionally, the NRSR at T1 and T2, and NRSW at T1 to T4 were positively correlated with body mass index (all *p* < 0.05).

**Conclusions:**

Serum inflammatory cytokines and muscle damage markers are positively correlated with acute postoperative pain following primary TKA, and the key cytokines (CRP, PGE2, and IL-6) and markers (Mb, CK and LDH) may serve as the targets for developing novel analgesic strategies.

**Electronic supplementary material:**

The online version of this article (doi:10.1186/s12891-017-1597-y) contains supplementary material, which is available to authorized users.

## Background

A growing prevalence of knee osteoarthritis (OA), one of the most common age-related knee diseases characterized by cartilage degradation leading to progressive joint pain and disability [1, 2], is expected with the aging of population, and various interventions, aiming at alleviating pain and improving joint function and quality of life, have been developing for OA treatment [3–5]. Total knee arthroplasty (TKA) is one of the most frequently performed surgical procedures, and the incidence of postoperative pain to vary degrees is also expected [6, 7]. Indeed, acute postoperative pain, being defined as an expected physiological response to surgery, is the most common and predicted problem following TKA [8, 9]. High level of acute postoperative pain have deleterious effects on individuals, it impedes short- and long-term functional rehabilitation, extends length of hospital stay and increases the risk of chronic postoperative pain [10–12]. Despite lots of original studies and guidelines regarding pain management have been reported, postoperative pain management following primary TKA is far from optimal and TKA is considered among the most painful surgeries [12–14]. Therefore, exploring the possible contributing factors and how relevant the factors are to the acute postoperative pain will be helpful for treatment modalities and development of new analgesic strategies.

It is commonly known that the body mass index (BMI), gender and surgical approach are associated with acute postoperative pain after TKA [15–19], and many studies reported that the serum inflammatory cytokines, including erythrocyte sedimentation rate (ESR), C-reactive protein (CRP), prostaglandin E2 (PGE2), interleukins (ILs), and tumor necrosis factor alpha (TNF-α), might also be related to postoperative pain [20–24], but few studies have detected the levels of the inflammatory cytokines in synovial fluid. Meanwhile, some researchers have used serum muscle damage markers, including myoglobin (Mb), creatinine (Cre), creatine kinase (CK), lactate dehydrogenase (LDH) and aspartate transaminase (AST), to compare different approaches in orthopedic surgeries [25, 26], but whether these markers were associated with acute postoperative pain following primary TKA is less known. Moreover, few studies have quantitatively analyzed the possible correlations between inflammatory cytokines, muscle damage markers and acute postoperative pain after primary TKA.

To address these questions, we first examined the serial changes of acute postoperative pain scores, serum and synovial fluid inflammatory cytokines, and serum muscle damage markers in patients with severe knee OA before surgery and in subsequent periods after primary TKA in this study. Then, we quantitatively investigated whether the inflammatory cytokines and muscle damage markers were significantly correlate with acute postoperative pain.

## Methods

### Study participants

From October 2014 to December 2015, consecutive patients were evaluated for primary knee OA and scheduled for a TKA at the Department of Orthopedic Surgery, West China Hospital, Sichuan University. Patients eligible for this study should be: (1) more than 18 years old, and able to understand the nature of this study (informed consent); (2) without any infectious, psychiatric or neurologic pathologies (e.g., psychosis and dementia); (3) with a diagnosis of OA with a severity grade ≥ 3 according to the Kellgren-Lawrence (KL) classification [27], and with a varus or valgus angle ≤30°; (4) undergoing elective, unilateral and primary TKA. Patients who met the following conditions were excluded: (1) allergy to nonsteroidal anti-inflammatory drugs (NSAIDs) because parecoxib and celecoxib would be sequentially administrated after surgery (described below); (2) history of gastrointestinal ulcer or bleeding; (3) alcohol and medical abuse within the 3 months preceding the inclusion; (4) being treated with corticosteroids, or hyaluronic acid within 6 months preceding the study, or systemic NSAIDs within 15 days before the study; (5) with progressive serious comorbidities (such as AIDS, malignant tumor, or end-stage renal or liver diseases), or with other painful conditions, or on medication that could possibly confound the evaluation of pain. Those who do not follow the study scheme, take other analgesics, withdraw the informed content, and are diagnosed with postoperative infection or other complications that will confound the results would be dropped out.

All procedures performed in this study were approved by the Ethical Committee of West China Hospital, Sichuan University, and with the 1964 Helsinki declaration and its later amendments or comparable ethical standards. Informed consent was obtained from all participants included in this study.

### Total knee arthroplasty and postoperative pain management

All patients were classified according to the ASA (physical status classification of the American Society of Anesthesiologists) scoring system and underwent general anesthesia with same anesthesia protocols by a same senior anesthetist. All surgeries were performed by the same senior joint surgeon (Bin Shen), who had performed more than 1000 TKAs, through the standard medial parapatellar approach in the same laminar air flow operating room, and posterior stabilized total knee prosthesis system (DePuy, New Jersey, USA) was used in all included patients. A pneumatic tourniquet was used in all patients, inflating before skin incision and releasing after prosthesis placement, and a suction drainage was indwelled before suture and removed on the first morning after surgery. All patients began ambulation on the postoperative day (POD) 1. As an alternative multimodal analgesic protocols, all patients were sequentially treated with intravenous parecoxib (40 mg/q12h), the first injectable cyclooxygenase-2 (COX-2) selective inhibitor with approved indication of postoperative pain [28], for the first three postoperative days and followed by oral celecoxib (200 mg/q12h) which could relieve pain superior to low doses of morphine (4 mg iv) for further analgesia [29]. Tramadol (100 mg/tablet, per os) or dolantin (10 mg, intramuscular injection) was used as rescue analgesic when the NRS at rest ≥4 [30].

### Pain assessment

Joint pain at rest (supine) and at walking (5 min after walk) were assessed 24 h before (T0), 24 h after (POD1, T1), 48 h after (POD2, T2), 72 h after (POD3, T3) and 5 days (POD5, T4) after surgery by 2 investigators using the numerical rating scale (NRS, with 0 represents no pain, and 10 represents the worst possible pain) [31]. Pain disability status was also assessed using the Pain Disability Questionnaire (PDQ), which is well validated in patients with musculoskeletal disorders compared with normal asymptomatic participants [32, 33]. The PDQ include 9 functional status items and 6 psychosocial items, and each was answered on a scale from 0 to 10, with 0 representing no disability and 10 representing maximal disability.

### Quantification of inflammatory cytokines and muscle damage markers

Venous blood samples were collected at T0 - T4, and synovial fluid specimens were collected before exposure of the capsule intraoperatively (considered as preoperative sampling). The levels of inflammatory cytokines, including ESR, CRP, PGE2, IL-6, IL-8 and TNF-α, and muscle damage markers, including Mb, Cre, CK, LDH and AST, in serum were measured by the Department of Laboratory Medicine of our hospital certified by the College of American Pathologists. Additionally, PGE2, IL-6, IL-8 and TNF-α in synovial fluid were detected as well.

### Statistical analysis

The demographics, operative information, NRS scores, values of inflammatory cytokines and muscle damage markers were collected. All statistical analyses were performed using the Statistical Package for Social Sciences (SPSS) software for Windows, version 22.0 (IBM, New York, USA). All continuous data were checked for normality first using Kolmogorov-Smirnov and Shapiro-Wilk tests. Comparisons between two groups (male and female) were conducted using the Student *t*-test for normally distributed data and Mann-Whitney *U*-test for non-normally distributed data, while comparisons among three groups (obese, overweight, and normal weight) were conducted using the one-way analysis of variance (ANOVA) with *post-hoc* analysis for normally distributed data, while Kruskal-Wallis *H* and Mann-Whitney *U* tests for non-normally distributed data. Spearman’s rank-based correlation coefficient was used to quantify the correlations between inflammatory cytokines, muscle damage markers and pain scores, as well as between BMI and pain scores. A *p*-value <0.05 was considered to indicate statistical significance. Since 15 markers (PGE2, IL-6, IL-8 and TNF-α were evaluated in both the serum and synovial fluid) were assessed, the statistical significance for multiple correlations between inflammatory cytokines, muscle damage markers and pain scores were further evaluated using the conservative Bonferroni *p* value (0.05/15 = 0.003) to control the family-wise error rate.

## Results

**Fig. 1:**
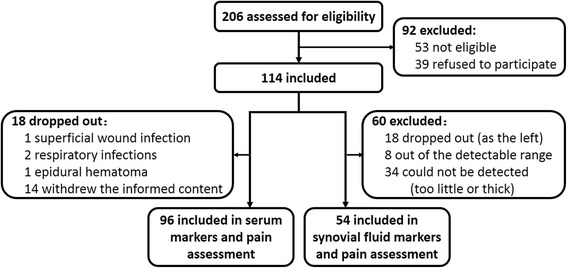
Flow diagram of patients through the phases of the study

Fig. 1 provides the full details of the study flow, 96 patients completed the study and included in the final analyses, and the raw data were shown in the Additional file 1. The overall mean age of the population was 65.96 ± 5.44 years with a mean body mass index (BMI) of 25.68 ± 2.80 kg/m^2^. The mean ASA was 1.75 ± 0.68, incision length was 15.21 ± 0.62 cm, operative time (from skin incision to the end of skin suture) was 58.20 ± 4.40 min, and the length of postoperative hospital stay (LPHS) was 7.11 ± 1.84 days. There was no significant differences in these characteristics between male (*n* = 18) and female (*n* = 78) patients. Additionally, synovial fluid inflammatory cytokines were detected in 54 patients (56.25%, Fig. 1).

### Correlations between pre- and postoperative pain

**Fig. 2:**
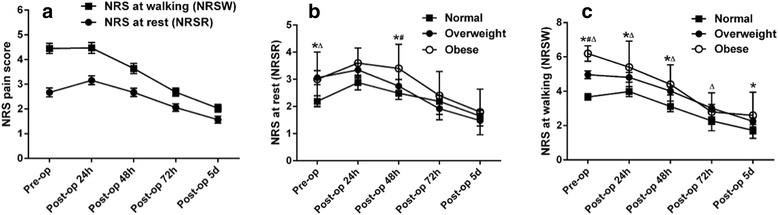
The serial changes of NRS pain scores after primary total knee arthroplasty (Mean ± 95% confidence intervals). **a** The serial changes of total NRS at rest and at walking following primary TKA. **b, c** Comparisons of NRS at rest (**b**) and at walking (**c**) among obese, overweight, and normal weight patients. *, the difference between obese and overweight patients was significant, *p* < 0.05; #, the difference between obese and normal weight patients was significant, *p* < 0.05; △, the difference between overweight and normal weight patients was significant, *p* < 0.05

The serial changes of NRS pain scores following primary TKA are shown in Fig. 2a, and there was no significant differences in NRS, both at rest (NRSR) and at walking (NRSW), between male and female patients. However, obese patients (BMI ≥ 30 kg/m^2^ [34], *n* = 5) tended to report higher NRS than overweight (25 ≤ BMI < 30 kg/m^2^, *n* = 48) and normal weight (BMI < 25 kg/m^2^, *n* = 43) patients, although not all of the differences were statistically significant (Fig. 2b and c). Associations between pre- and postoperative pain scores are shown in Table 1. Postoperative NRSW at T1 to T4 were positively correlated with preoperative NRSR, NRSW and PDQ (all *p* < 0.05), while postoperative NRSR at T1 and T2 were positively correlated with preoperative NRSW (all *p* < 0.001). These results remained significant even if after adjusting for multiple comparisons (Bonferroni *p* value, 0.05/3 = 0.017). Furthermore, the preoperative NRSW was positively correlated with NRSR and PDQ (*r* = 0.30, *p* = 0.003; *r* = 0.40, *p* < 0.001, respectively), while no significant correlation between preoperative NRSR and PDQ was found.

**Table 1:** Correlations between pre- and postoperative pain, *n* = 96 (*Spearman’s correlation coefficients, p*)

Pre-op scores	Post-op NRSR				Post-op NRSW			
	T1	T2	T3	T4	T1	T2	T3	T4
Pre-op NRSR	0.01, 0.911	0.04, 0.706	−0.14, 0.182	−0.07, 0.482	0.34, 0.001*	0.37, <0.001*	0.25, 0.013*	0.26, 0.010*
Pre-op NRSW	0.40, <0.001*	0.37, <0.001*	−0.001, 0.995	−0.06, 0.595	0.30, 0.003*	0.38, <0.001*	0.39, <0.001*	0.34, 0.001*
Pre-op PDQ	0.08, 0.432	0.06, 0.587	−0.12, 0.240	0.04, 0.697	0.75, <0.001*	0.52, <0.001*	0.47, <0.001*	0.36, <0.001*

*Abbreviations: NRSR* numerical rating scale at rest, *NRSW* numerical rating scale at walking, *PDQ* pain disability questionnaire, *T1-T4* 1, 2, 3 and 5 days after surgery, respectively. The significant correlations according *p* < 0.05 (*) remained significant after Bonferroni correction (*p* = 0.05/3).

### Correlations between inflammatory cytokines and acute postoperative pain

**Fig. 3:**
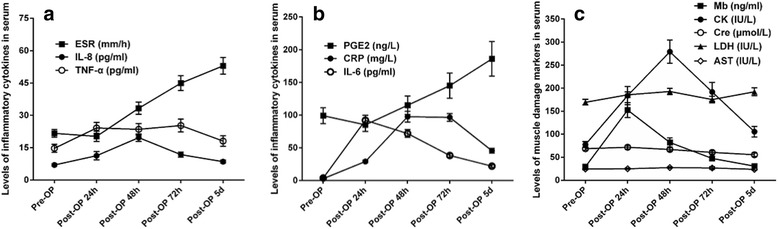
The serial changes of serum inflammatory cytokines and muscle damage markers after primary total knee arthroplasty. **a, b** The serial changes of serum inflammatory cytokines, including erythrocyte sedimentation rate (ESR); C-reactive protein (CRP), prostaglandin E2 (PGE2), interleukin-6 and -8 (IL-6 and IL-8), tumor necrosis factor alpha (TNF-α). **c** The serial changes of serum muscle damage markers, including myoglobin (Mb), creatinine (Cre), creatine kinase (CK), lactate dehydrogenase (LDH), and aspartate transaminase (AST)

The serial changes of serum inflammatory cytokines are shown in Fig. 3a and b, and the associations between serum inflammatory cytokines and pain scores are shown in Table 2. Before surgery (at T0), the NRSR was positively correlated with ESR and IL-6; the NRSW was positively correlated with ESR, CRP, PGE2 and IL-6; and the PDQ was positively correlated with ESR, CRP and PGE2 (all *p* < 0.003, with Bonferroni correction). Postoperatively, the NRSR was positively correlated with PGE2 and IL-6 at T1; and the NRSW was positively correlated with CRP at T1, and with PGE2 and IL-6 at T1 to T3 (all *p* < 0.003).

**Table 2:** Correlations between serum inflammatory cytokines and pain scores, *n* = 96 (*Spearman’s correlation coefficients, p*)

	PDQ	NRSR					NRSW				
		T0	T1	T2	T3	T4	T0	T1	T2	T3	T4
ESR	0.31, 0.002*	0.44, <0.001*	0.04, 0.691	−0.13, 0.203	−0.04, 0.718	−0.10, 0.339	0.53, <0.001*	0.13, 0.192	0.15, 0.142	0.04, 0.670	0.04, 0.672
CRP	0.37, <0.001*	0.24, 0.017	0.04, 0.689	−0.08, 0.446	0.00, 0.982	−0.02, 0.814	0.34, 0.001*	0.38, <0.001*	0.29, 0.004	0.15, 0.134	−0.03, 0.743
PGE2	0.44, <0.001*	0.24, 0.017	0.37, <0.001*	0.29†, 0.005	0.06, 0.584	−0.02, 0.824	0.67, <0.001*	0.35, 0.001*	0.42, <0.001*	0.39, <0.001*	0.29, 0.004
IL-6	0.26, 0.009	0.34, 0.001*	0.31, 0.002*	0.28, 0.007	−0.07, 0.474	−0.20, 0.047	0.33, 0.001*	0.41, <0.001*	0.40, <0.001*	0.37, <0.001*	0.22, 0.034
IL-8	-0.05, 0.641	−0.10, 0.314	−0.13, 0.224	−0.07, 0.520	−0.20, 0.055	−0.04, 0.709	−0.03, 0.782	−0.17, 0.107	−0.24, 0.020	0.21, 0.041	−0.07, 0.937
TNF-α	0.09, 0.404	0.16, 0.110	−0.01, 0.907	−0.14, 0.186	−0.11, 0.302	−0.06, 0.583	0.18, 0.080	0.05, 0.619	0.06, 0.576	−0.01, 0.944	−0.04, 0.737

*Abbreviations: NRSR* numerical rating scale at rest, *NRSW* numerical rating scale at walking, *PDQ* pain disability questionnaire, *ESR* erythrocyte sedimentation rate; *CRP* C-reactive protein, *PGE2* prostaglandin E2, *IL* interleukin, *TNF-α* tumor necrosis factor alpha, *T0* 24 h before surgery, *T1-T4* 1, 2, 3 and 5 days after surgery, respectively. The *p*-values are presented in their raw, uncorrected form, but the statistical significance for multiple comparisons were further corrected using the conservative Bonferroni *p* value (0.05/15 = 0.003) to control the family-wise error rate, and *p* < 0.003 are deemed significant (*).

The overall mean values of preoperative PGE2, IL-6, IL-8 and TNF-α in synovial fluid (*n* = 54) were 43.73 ± 17.85 ng/ml, 128.29 ± 104.2 pg/ml, 41.74 ± 21.58 pg/ml and 22.44 ± 11.34 pg/ml, respectively. Associations between synovial fluid inflammatory cytokines and pain scores are shown in Table 3. Preoperatively, the NRSW was positively correlated with PGE2, IL-6, IL-8 and TNF-α; the PDQ was positively correlated with PGE2 (all *p* < 0.003); while no significant correlations between preoperative NRSR and cytokines was found (all *p* ≥ 0.003). Furthermore, there was also no significant correlations between postoperative NRS, both NRSR and NRSW at T1 to T4, and preoperative synovial fluid cytokines (all *p* ≥ 0.003). Additionally, the preoperative PGE2, as well as IL-6, in serum and synovial fluid were also positively correlated (*r* = 0.54, *p* < 0.001; and *r* = 0.33, *p* = 0.014, respectively).

**Table 3:** Correlations between synovial fluid inflammatory cytokines and pain scores, *n* = 54 (*Spearman’s correlation coefficients, p*)

	PDQ	NRSR					NRSW				
		T0	T1	T2	T3	T4	T0	T1	T2	T3	T4
PGE2	0.43, 0.001*	0.08, 0.588	0.38, 0.004	0.36, 0.007	0.03, 0.806	0.15, 0.286	0.52, <0.001*	0.33, 0.017	0.29, 0.032	0.26, 0.055	0.14, 0.317
IL-6	0.34, 0.011	0.08, 0.577	0.17, 0.225	0.09, 0.542	−0.22, 0.115	−0.12, 0.409	0.47, <0.001*	0.31, 0.021	0.27, 0.051	0.25, 0.065	0.07, 0.625
IL-8	0.15, 0.264	0.32, 0.019	0.10, 0.464	0.04, 0.763	−0.16, 0.254	−0.21, 0.122	0.74, <0.001*	0.16, 0.235	0.22, 0.109	0.20, 0.157	0.13, 0.350
TNF-α	0.28, 0.043	0.30, 0.029	0.19, 0.159	0.30, 0.028	−0.05, 0.740	−0.15, 0.295	0.76, <0.001*	0.25, 0.069	0.22, 0.116	0.22, 0.111	0.26, 0.062

*Abbreviations: NRSR* numerical rating scale at rest, *NRSW* numerical rating scale at walking, *PDQ* pain disability questionnaire, *PGE2* prostaglandin E2, *IL* interleukin, *TNF-α* tumor necrosis factor alpha, *T0* 24 h before surgery, *T1-T4* 24, 48, 72 h and 5 days after surgery, respectively. The *p*-values are presented in their raw, uncorrected form, but the statistical significance for multiple comparisons were further corrected using the conservative Bonferroni *p* value (0.05/15 = 0.003) to control the family-wise error rate, and *p* < 0.003 are deemed significant (*)

### Correlations between muscle damage markers and acute postoperative pain

The serial changes of serum muscle damage markers are shown in Fig. 3c, and the correlations between serum muscle damage markers and pain scores are shown in Table 4. Preoperative PDQ was positively correlated with Cre (*p* = 0.001), while no correlations between NRS and muscle damage markers was observed (all *p* ≥ 0.003). Postoperatively, the NRSR was positively correlated with CK at T1 (*p* < 0.001); the NRSW was positively correlated with Mb and LDH at T1 to T4, and with CK at T2 and T4 (all *p* < 0.003).

**Table 4:** Correlations between serum muscle damage markers and pain scores, *n* = 96 (*Spearman’s correlation coefficients, r*)

	PDQ	NRSR					NRSW				
		T0	T1	T2	T3	T4	T0	T1	T2	T3	T4
Mb	0.19, 0.059	0.28, 0.006	0.11, 0.278	0.06, 0.535	0.04, 0.688	0.16, 0.121	0.20, 0.051	0.46, <0.001*	0.32, 0.001*	0.36, <0.001*	0.35, <0.001*
Cre	0.33, 0.001*	0.09, 0.398	−0.12, 0.981	0.16, 0.111	0.22, 0.028	−0.05, 0.653	0.07, 0.531	−0.16, 0.110	−0.11, 0.297	−0.09, 0.409	0.07, 0.475
CK	0.06, 0.554	0.27, 0.007	0.39, <0.001*	0.14, 0.189	−0.02, 0.850	0.01, 0.910	0.25, 0.014	0.24, 0.019	0.34, 0.001*	0.29, 0.004	0.34, 0.001*
LDH	0.23, 0.022	0.26, 0.011	0.25, 0.016	0.10, 0.330	0.04, 0.683	−0.07, 0.513	0.30, 0.003	0.32, 0.001*	0.41, <0.001*	0.47, <0.001*	0.36, <0.001*
AST	0.12, 0.240	0.21, 0.039	0.07, 0.504	0.001, 0.991	−0.002, 0.986	−0.13, 0.198	0.25, 0.013	0.09, 0.399	0.10, 0.314	0.28, 0.005	0.18, 0.075

*Abbreviations: NRSR* numerical rating scale at rest, *NRSW* numerical rating scale at walking, *PDQ* pain disability questionnaire, *Mb* myoglobin, *Cre* creatinine, *CK* creatine kinase, *LDH* lactate dehydrogenase, *AST* aspartate transaminase, *T0* 24 h before surgery, *T1-T4* 1, 2, 3 and 5 days after surgery, respectively. The *p*-values are presented in their raw, uncorrected form, but the statistical significance for multiple comparisons were further corrected using the conservative Bonferroni *p* value (0.05/15 = 0.003) to control the family-wise error rate, and *p* < 0.003 are deemed significant (*)

### Correlations between BMI and acute postoperative pain

Because the obese patients tended to report higher NRS than overweight and normal weight patients (see above), the correlations between BMI and pain scores were further evaluated. As a result, preoperative NRSR, NRSW and PDQ were positively correlated with BMI (*r* = 0.56, 0.80, and 0.37, respectively; and all *p* < 0.001). Meanwhile, the NRSW at T1 to T4 (*r* = 0.80, 0.44, 0.53, and 0.44, respectively; and all *p* < 0.001), as well as the NRSR at T1 and T2 (*r* = 0.56, *p* = 0.001; and *r* = 0.26, *p* = 0.010, respectively), were also positively correlated with BMI.

## Discussion

TKA is a successful procedure aiming to ultimately alleviate pain and restore joint function in patients suffering from end-stage knee diseases [1, 32, 35]. The management of acute postoperative pain, however, is still far from optimal, and TKA is among the most painful surgeries [19, 36]. In this study, we quantitatively assessed the possible correlations between inflammatory cytokines, muscle damage markers and acute postoperative pain, which few studies have reported, in 96 consecutive primary TKA patients. As a result, we found that the rest pain of the operated knee was positively correlated with serum PGE2, IL-6, and CK at POD 1; while the walking pain was positively correlated with serum CRP at POD 1, with PGE2 and IL-6 at POD 1 to POD 3, with CK at POD 2 and POD 5, and with Mb and LDH at POD 1 to POD 5, indicating that some inflammatory cytokines (CRP, PGE2, IL-6) and muscle damage markers (Mb, CK, and LDH) are indeed interacted with acute postoperative pain after primary TKA.

Inflammatory cytokines, including PGE2, ILs (mainly IL-6) and TNF-α, presented in serum and synovial fluid in OA pathogenesis [37, 38], and it raises the question that whether these cytokines associated with acute postoperative pain after TKA. It has been proved that surgery and trauma are often accompanied by changes in serum levels of certain cytokines and markers, including inflammatory cytokines and muscle damage markers [39, 40]. However, to our knowledge there is no studies investigated the associations between these markers and acute postoperative pain following primary TKA. Large amounts of IL-6 are produced at the surgical site and enter the systemic circulation, where its concentrations correlate with the severity of surgery and the magnitude of the tissue injury [41]. After surgery or injury, IL-6 levels were detectable in the systemic circulation at 60 min, it reached to a peak value between 4 to 6 h, and could persist for as long as 10 days [20]. We were not able to specify the exact time of peak value of IL-6 in this study, but we found that the IL-6 value was significant elevated within the first postoperative day and then began to decline. We also observed that the serum IL-6 levels were positively correlated with the acute postoperative pain, with rest pain at POD 1 and with walk pain at POD 1 to POD 3, following primary TKA. Similar correlations was also found between acute postoperative pain and PGE2, which could (indirectly) increase the sensitization of nociceptors and acute postoperative pain [38]. TNF-α has been shown to influence and coordinate the inflammatory response in almost all tissues, it can influence the excitability of nociceptors either directly or through the expression of downstream cytokines, or both, and play a crucial role in the pathophysiology of injury-related pain. However, no significant correlations between acute postoperative pain and TNF-α, both in serum and in synovial fluid, was found in our study. Additionally, it would be specially mentioned that we initially detected the IL-1β, which was reported as a strong pro-inflammatory cytokine [42, 43], but its level was very low and difficult to be detected in the systemic circulation, as well as in synovial fluid, even in subsequent periods following surgery, and this was also been reported by other researchers [24, 44].

ESR is a non-specific hematological marker routinely used as an indirect parameter of increased acute phase reactants, and CRP is a major acute phase reactant and produced by the liver in response to inflammation, infection, malignancy or tissue damage [23]. Honsawek et al. reported that ESR was increased with a peak value reached 2 weeks after TKA and reduced to its preoperative level at 26 weeks postoperatively, while the CRP was elevated on the POD 1 and decreased to its preoperative value at 2 weeks postoperatively [24]. However, this study showed that the values of ESR were slightly declined on POD 1, and then evaluated with a continuously slow rise, while the CRP values were increased to peak on POD 2, and then began to decline. We further found that the postoperative walking pain at POD 1 were positively correlated with the CRP, but no correlation between postoperative pain and ESR was found. Overall, these results demonstrate that acute postoperative pain after TKA was indeed associated with inflammatory cytokines, in which CRP, PEG2, and IL-6 are the key ones.

We further quantitatively analyzed the correlations between muscle damage markers and acute postoperative pain following primary TKA, and identified that Mb, CK and LDH were the key markers in systemic circulation which positively correlated with acute postoperative pain. Muscle damage could occurred not only intraoperatively, but also postoperatively. The medial parapatellar retinacular incision and release of medial collateral ligament (MCL) were inevitable in TKA, and contribute to the intraoperative muscle damage. Niki et al. reported that the levels of muscle-related enzymes were affected by the degree of medial release for appropriate soft-tissue balancing [26]. Despite the medial collateral ligament (MCL) and semimembranosus muscle were intraoperatively released from the tibia by subperiosteal dissection, which could avoid damage to the actual muscle fibers theoretically, Niki et al. reported that such release appear to elevate muscle damage marker levels [26]. Meanwhile, postoperative functional exercise, including walking and flexing, in the early stage after surgery might also cause potential muscle fiber injury and subsequent elevations in relative markers in serum. Additionally, it is still debated whether tourniquet-induced ischemia represents a substantial contributor to inflammatory response and muscle damage, as well as subsequent release of relative markers. Clementsen et al. investigated the release of inflammatory cytokines, including IL-1β, IL-6, IL-8, and TNF-α, after tourniquet use in TKA, but levels of these cytokines did not change regardless of tourniquet use [44]. Laurence et al. evaluated tourniquet-induced ischemia during TKA and reported that no significant difference in Mb between patients with and without tourniquet (less than 150 min), and the elevation of serum Mb associated with tourniquet was negligible [45].

In this study, obese patients tended to report higher pain scores than overweight and normal weight patients, and the rest pain at POD1 and POD2, as well as the walking pain at POD1 to POD 5, were positively correlated with BMI. Although these results further supported our previous finding that a BMI ≥ 30 kg/m^2^ have a negative influence on the outcomes of primary TKA [34], but whether obese patients should be encouraged to lose weight before TKA is still controversial, and many other factors, such as age, physical condition and the feasibility of losing weight, should be taken into account [46]. Moreover, more than half of the patients included in this study with a BMI ≥ 25 kg/m^2^ (*n* = 53), in which only 5 were obese, the unbalanced sample size might introduce a bias, and further studies with more subjects and appropriate BMI constitution were needed to further illustrate the effects of BMI on acute postoperative pain after primary TKA.

Additionally, although the overall walking pain was more severe than rest pain, some patients reported a more severe rest pain than walking pain in the same day. There is only one relative study, by Lunn et al., reported that acute postoperative pain at rest and during hip and knee flexion after TKA was significantly reduced 5 min and 20 min after walk compared with that before walk, and pain was further reduced during the second walk compared with the first walk [47]. We assessed NRSW 5 min after walk and similar results were obtained in partial patients, indicating that mobilization might be able to promote analgesic effects. Future studies with a randomized, controlled design on exercise dose-response effects after primary TKA were necessary to illustrate this phenomenon.

However, several limitations must be taken into account in this study. Firstly, the subjects included in this study were confined to OA patients, and therefore it could not be generalized to other types of knee arthrosis, such as rheumatoid arthritis. Secondly, only 54 out of 96 (56.25%) patients had the synovial fluid data collected for preoperative analyses because the synovial fluid in some patients was too little or viscous and could not be detected, and the postoperative synovial fluid cytokines were also not assessed because the drainage tube was removed on the first morning after surgery. Thirdly, no control group, such as unicompartmental knee arthroplasty or minimally invasive approach, was established, and the joint function was not reported in this study. Finally, other confounding factors, such as fear or anxiety, articular nerves, implant type, and anticoagulant used for prophylaxis of postoperative venous thromboembolism [48–50], might also affected the acute postoperative pain following TKA.

## Conclusions

We demonstrate in this study that serum inflammatory cytokines and muscle damage markers are positively correlated with acute postoperative pain following primary TKA, and the key serum cytokines (CRP, PGE2, and IL-6) and markers (Mb, CK and LDH) may serve as the targets for developing novel analgesic strategies.

## Additional file

Additional file 1: File name: The raw data.xlsx. Title of data: The raw data of each participant. Description of data: The data include the general and operative information, PDQ and NRS scores, serum inflammatory cytokines and muscle damage markers levels, and synovial fluid inflammatory cytokines levels of each participant. (XLSX 49 kb)

## Abbreviations

AST: Aspartate transaminase
BMI: Body mass index
Cre: Creatinine
CK: Creatine kinase
CRP: C-reactive protein
ESR: Erythrocyte sedimentation rate
IL: Interleukin
LDH: Lactate dehydrogenase
Mb: Myoglobin
NRS: Numerical rating scale
NRSR: NRS at rest
NRSW: NRS at walking
PDQ: Pain disability questionnaire
PGE2: Prostaglandin E2
POD: Postoperative day
TKA: Total knee arthroplasty
TNF-α: Tumor necrosis factor alpha
